# CICing the partnership model into the future: a new social enterprise model for general practice?

**DOI:** 10.3399/BJGP.2025.0326

**Published:** 2025-07-22

**Authors:** Adrian Crofton, Cam Donaldson

**Affiliations:** 1 Torry Medical Practice, River Dee Medical Group, Aberdeen, UK; 2 Glasgow Caledonian University, Glasgow, UK; 3 Australian National University, Canberra, Australia

For some years, the number of new GPs going into partnership across the whole of the UK has been decreasing, while the number of GPs exiting partnerships has been increasing.^1,2^ These trends show no sign of reversing and there are no longer enough partners to sustain many partnerships. There are a number of causes, including continued NHS austerity, the COVID-19 pandemic, and changing workforce patterns, with growing numbers of GPs opting for salaried and part-time working.^1–3^ There is an urgent need, therefore, across all four nations of the UK to identify viable alternative ownership models for primary care. Given the central role played by general practice in the NHS, this needs to happen before a tipping point is reached from which it would be difficult to return.

## The partnership crisis

The partnership crisis has led to structural responses in primary care that are often maladaptive. The creation of conglomerates is a natural market response to austerity, with some practices being put straight to tender and others after a period of direct NHS management. Conglomerates might provide economies of scale while conveniently allowing government to claim that populations remain covered. However, such ownership models might also be damaging to the overall effectiveness of the health and social care system. They are often not based within the localities they purport to serve, and manage budget restraints through scale efficiencies that lead to care that is increasingly modularised, transactional, and lacking continuity. The cumulative effect of this is to offset costs to other parts of the health and social care system. As such, it is unclear how such models fit with government aspirations of greater integration across health and social care. This fragmentation of care also passes on more costs to patients, particularly in terms of access, and can disproportionately impact those with the greatest health needs, further reinforcing existing health inequalities.

At this juncture, it is important and timely to ask whether there are other alternatives that, ideally, retain the best aspects of the partnership model while also offering something more. The virtues of partnerships are their independence accompanied by flexibility, versatility, team identity, and continuity of care for patients (itself one of the greatest determinants of good health outcomes).

*“Given the acknowledged need for a new vision for general practice and primary care as a whole, CICs are a natural nidus for this: accountability combined with whole-system efficiency, allowing clinicians to work effectively at scales that are both locally responsive but also large enough to facilitate reliable and comprehensive clinical cover at all times.”*

## Community Interest Companies

Community Interest Companies (CICs) are a legal form of social enterprise that offer precisely this. As locally-based independent contractors, CICs retain key features of the partnership system, but also have additional benefits for both patients and staff. These include greater financial transparency, local accountability, and benefits for professionals such as appropriate economies of scale to allow for cross-cover and potential for improved career structure. It also allows for a more transparent and unified salary scale that may enable GPs to begin to address the general decline in income, but also of terms and conditions, relative to secondary care colleagues. Mechanisms such as ‘asset locks’ and public access to company accounts ensure that surplus income is reinvested in the service and that ownership remains within the local community. The fourth Social Enterprise Census in Scotland reveals a thriving and growing model of working.^4^ There are now over 6000 social enterprises in Scotland, contributing to many sectors of economic activity across the country and winning contracts from the public sector based on their social impact. There is a growing evidence base demonstrating the abilities of CICs to develop bespoke rehabilitation programmes, focus on those in greatest need, and develop strong community partnerships in doing so.^5,6^ Positive case studies of social enterprises in delivery of community-based health services are now being reported and they are also the subject of further rigorous evaluation.^7,8^

As such, CICs have clear advantages over alternative ownership models, namely the quasi- or fully privatised ‘super-practices’ but also over direct NHS ownership. Given the acknowledged need for a new vision for general practice and primary care as a whole, CICs are a natural nidus for this: accountability combined with whole-system efficiency, allowing clinicians to work effectively at scales that are both locally responsive but also large enough to facilitate reliable and comprehensive clinical cover at all times. Despite recently announced plans to invest small amounts to modernise general practice facilities,^9^ the environment remains one of persistent underfunding^10,11^ in which no ownership model can itself provide a panacea. However, sole reliance on traditional ownership models that have formed the bedrock of general practice in the past increases the risk of sudden system failure while losing the additional opportunities offered by these alternative models. Looking to the future, we propose a rethink of the concept of ownership in general practice and primary care, employing a greater and more imaginative range of proven alternatives.
